# Knowledge Mapping of Enterprise Network Research in China: A Visual Analysis Using CiteSpace

**DOI:** 10.3389/fpsyg.2022.898538

**Published:** 2022-06-30

**Authors:** Wancheng Yang, Shaofeng Wang, Chen Chen, Ho Hon Leung, Qi Zeng, Xin Su

**Affiliations:** ^1^Logistics and e-Commerce College, Zhejiang Wanli University, Ningbo, China; ^2^Smart Learning Institute, Beijing Normal University, Beijing, China; ^3^School of Humanities and Communication, Ningbo University, Ningbo, China; ^4^SUNY Oneonta, Oneonta, NY, United States; ^5^International Exchange College, Zhejiang Business Technology Institute, Ningbo, China; ^6^School of Economics and Management, Beijing University of Posts and Telecommunications, Beijing, China

**Keywords:** enterprise network, knowledge mapping, visual analysis, CSSCI, CiteSpace

## Abstract

Enterprise Network (EN) has increasingly gained popularity in academia. Over the past few decades, a substantial amount of EN studies have been published in China. Drawing upon a sample of 603 papers retrieved from the Chinese Social Sciences Citation Index database (CSSCI) between 1998 and 2020, this study aims to delve into the status quo, knowledge base, research focus, and evolutionary trends of EN research in China. A multifaceted bibliometric analysis was performed using CiteSpace. The findings mainly indicate that the research on EN in China has a clear development context, and the research content gradually changes from macro to micro. In addition to foreign Social Network theories, the research results of domestic scholars have become the basic knowledge in this field. The research includes these topics: the conceptualization of EN, EN as indicators of enterprise development, EN’s impact on start-ups, mechanisms of EN’s effect and governance of EN. The potential direction for future research has been identified as the integration between EN and other forms of networks, and the structure of EN.

## Introduction

Since the 1980s, with social network research methods in economic management, Enterprise Network (EN) has attracted extensive attention in practice and theory. EN is the social networks among organizations or enterprises (Powell et al., 1996) or the connections of different parts bound by certain social relations (Laumann et al., 1978). *Social networks* are defined as a relatively stable system of individuals and their social relations (Wellman, 1988). Social network theories emerged as a new paradigm for sociological studies (Maltseva and Batagelj, 2021), which embodied two major analytical factors: relation and structure (Li et al., 2021). As the research scope extended further, social network theories had reached beyond the level of interpersonal relations and increasingly been exerting a profound influence on enterprise knowledge, information, and other forms of resources (Asamoah et al., 2020; Maltseva and Batagelj, 2020; Busch and Barkema, 2022). Specifically, the concept of “embeddedness” proposed by Mark Granovetter has addressed the influence of social networks on economic behaviors, suggesting that individuals are “embedded” in their social networks. Hence they are unavoidably affected by their social relations (Granovetter, 1985). As propounded by Granovetter, humans, as the core of economic behaviors, regardless of their distinct attributes, other members of their social networks within which information and resources are embedded tend to impact their decision-making (Sánchez-Arrieta et al., 2021). Such a pattern also applies to the organizational level; the interplay between enterprises and other organizations has shaped different forms of connections. Such connections are “embedded” within their social networks, which have become a key source of funds, know-how, and techniques (Li and Jia, 2014; Chang and Wang, 2016; Kebede, 2020; Yu et al., 2021; Chen et al., 2022).

Influenced by Confucianism, Chinese business managers, use interpersonal relationships (such as family, classmates, communities, and colleagues) as social network resources to help them obtain business information and advantages (Sanders and Nee, 1996; Iyer and Shapiro, 1999; Chen, 2004). In the relational culture (Wang et al., 2015; Geng et al., 2017), EN is seen as a critical resource; it can improve the competitiveness of enterprises (Wang and Chung, 2013). It can be seen that although the EN theory originated from the Western scenario, China has become an excellent place to test and develop EN theory, as the market, financial and other formal systems have not yet been perfected, and the relational culture influences enterprise managers. Up to now, lots of Chinese scholars have conducted in-depth research on the concept, function, and governance of EN.

However, there is a lack of comprehensive, quantitative reviews exclusively focused on EN studies in China. Our work in this study provided an in-depth picture of the EN research’s status quo and evolution and predicted the future tendency in this field. Specifically, we analyzed 603 publications on EN from the Chinese Social Sciences Citation Index database (CSSCI) from 1998 to 2020. By analyzing bibliometric indicators achieved on CSSCI, we illustrated the distribution of publications, most influential journals, essential authors, and outstanding institutions. Furthermore, by analyzing data with CiteSpace, we presented the co-citation of cited references, cited authors, and cited journals. We also explored the evolution of the EN research by using the keyword clustering feature of CiteSpace. On this basis, we predicted the future tendencies in the EN research field.

This study has three contributions. First, it reflects the status quo and the content of EN research in China more directly, which makes it clear and easy to trace the origin in this field. Second, it shows the development trace in the EN research in China, which assists scholars in profoundly understanding the evolution in this field and then recognizing new directions. Third, it shows the most influential institutions, journals, and references in the EN research in China, which helps scholars accurately search for journals, authors, and papers.

Section “Literature review” of this paper presents the literature review on EN research. Section “Methodology” describes the bibliometric methods and the data for analysis in this study. Section “Results” presents the results of the bibliometric analysis, including the status quo, knowledge base, hot topics, and research trends of EN studies in China. Finally, We finish with a discussion of the results and some concluding remarks.

## Literature Review

Enterprise Network originates from social network research. T Wellman proposes the mature definition of the Social Network as “a relatively stable system constituted of individuals and their social relations” (Wellman, 1988), i.e., he notices “network” seen as a series of social connections or social relations. As the social network is applied to more fields, it has gone beyond the level of interpersonal relations. As Butler pointed out, market information provided by entrepreneurs’ social networks extensively influences entrepreneur behavior (Butler, 1991). Participants in social networks are no longer limited to individuals but can be a community, such as a family, department, organization, institute, or country. With its origin in sociology, the social network is now applied in fields like pedagogy and economics as relevant theories are further developed and improved. More and more scholars have developed the concept of “social structure,” and social network theories have gradually been recognized by scholars (Kereri and Harper, 2019).

There are two different views on the definition of Enterprise Network. Firstly, we investigated the Enterprise Network that maintains long-term relations in two or more enterprises, and it falls between the external free market and internal hierarchical governance (Powell et al., 1996). The other is to define EN as connections among different parts (such as individuals, enterprises, organizations, or countries) bound by certain social relations (such as transactional relations, blood relations, organizational relations, etc.) (Laumann et al., 1978). The network perspective breaks the limitation of explaining enterprise governance only from the perspective of individual behaviors (Chen and Gong, 2017). EN contains a wide variety of resources; enterprises can gain information and resource advantages by reaching out to entities in the network (Chuluun et al., 2017). Due to the impact of different uncertainties, businesses are focusing on developing individual partnerships with the help of EN to achieve competitive advantage (Anderson et al., 2019). EN can help entrepreneurs keep in touch with others or other organizations and obtain opportunities and resources (Suryandharu et al., 2019). EN also helps create an environment where individuals and businesses can share information for sustainable business development (Berraies, 2019; Ülgen, 2019). According to Li and Jia (2014) and Maltseva and Batagelj (2020), western research on social networks is mainly conducted from the structural and instrumental perspectives.

Although the EN research in China started later than in the West, a large number of research results have emerged in the specific context of China’s economic development. For example, Yu et al. (2020) pointed out that the EN contains rich knowledge elements, and enterprises actively affect innovation performance through knowledge absorption, knowledge link, and knowledge analysis ability. In addition, the EN formed by the personal social relations of corporate executives such as independent directors has also been proved to play a positive role in promoting corporate M & A performance (Liang et al., 2018), innovation performance (Wei and Chen, 2019), and technology acquisition (Li and Liu, 2021). Given its crucial role in initiating, sustaining, and expanding ventures, EN theories have increasingly drawn scholarly attention in business management studies, and a large number of research results have emerged. To analyze the status quo of EN studies in China, Xu and Xiang (2006) synthesized EN’s growth curve and anticipated three main directions for future research. Peng and Sheng, respectively, reviewed the literature regarding inter-organizational networks and entrepreneurship to depict EN’s evolution path (Peng and Wang, 2010; Sheng and Peng, 2010). Further, to address the problem of how such enterprise networks can be managed and the associated resources optimized in the context of enterprises being “embedded” within social networks, Liao (2007), Yu and Zhang (2010), and Li et al. (2014) drawing upon network governance theories, summarized the conceptual framework and research front of EN in China.

It is noteworthy that the previous papers are all based on qualitative approaches. There is a scarcity of quantitative studies surrounding EN in China. Until very recently, bibliometric analysis has been adopted by Chinese EN scholars. Among the limited number of studies, Zhou launched on a few factors such as authors, institutions, and research topics to appraise EN practice in China quantitatively (Zhou and Ruan, 2018). Nonetheless, it is rather difficult for such a limited number of quantitative factors to thoroughly disclose the knowledge base in the field. Correspondingly, the current status, research hotspots, and evolutionary trends can barely be precisely reflected, as the divergent research themes in the EN research field make the existing research chaotic. To gain a profound understanding of domestic EN’s status quo and progression, we conduct an integrative review through knowledge mapping analysis, which aims at drawing a holistic and systematic picture in this field. It becomes imperative for the present study to launch on a quantitative approach to map out the landscape of EN studies in China holistically.

## Methodology

### Knowledge Mapping Analysis

Knowledge maps, also known as knowledge domain maps, consist of various graphs showcasing the correlation between the process of knowledge progression and network structure. They are purposefully visualized to depict knowledge resources and the corresponding entities that maintain, and extract, analyze, synthesize, and present knowledge elements and interplay (Li and Li, 2018; Jaradeh et al., 2019). Apart from exhibiting how a knowledge element is relatively positioned within its disciplinary knowledge area, knowledge mapping also serves to represent the research frontier and pre-estimate the progressive trends of a discipline (Kastrin and Hristovski, 2021). Hence, it is extensively adopted across different disciplines including sociology, economics, and information science. Among the several knowledge mapping tools, CiteSpace remains frequently used in many prior works for data visualization and analysis (Zhu and Yang, 2018; Yang and Ning, 2021; Zhang et al., 2021). Given its wide application in academia, CiteSpace was employed in this study to facilitate the construction and visualization of knowledge mapping of the EN studies in China.

### Data Source

The Chinese Social Sciences Citation Index (CSSCI) is a high-quality digital database that has been broadly accepted among researchers in China; it was developed by the Chinese Social Sciences Research Evaluation Center based at Nanjing University. The CSSCI represents high-quality journal rankings in the academic field of China and covers a wide range of publications in social sciences. In addition, the CSSCI is a proper database because it contains a set of data, such as titles, authors, institutions, keywords, and references. Our study analyzed publications from 1998 to 2020, as CSSCI only includes studies from 1998 to the present day. Apart from “Enterprise Network,” we used different keywords to search pertinent publications. Having excluded literature irrelevant to EN or beyond the research scope of this study, we obtained a unique database that includes 603 publications. The database is a text file that includes the variables of title, author, publish year, abstract, keywords, and references.

### Procedures of CiteSpace Analysis

**FIGURE 1:**
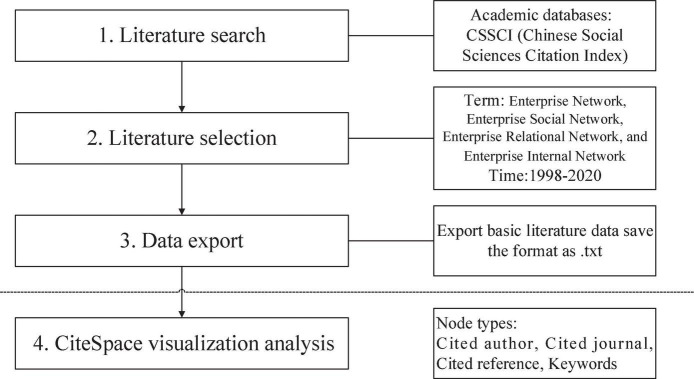
Flow diagram of the bibliometric analysis performed with CiteSpace.

CiteSpace 5.8.R2 was adopted to perform the knowledge mapping analysis of 603 CSSCI papers themed with “Enterprise Network” to investigate the status quo, knowledge base, research focus, and evolutionary trends of EN studies in China. The overall research process entails four phrases, as shown in Figure 1.

The Law of Bradford states that most papers are in a few acclaimed journals in the same area (Qiu, 2000). The literature in this study was retrieved from CSSCI (Chinese Social Sciences Citation Index) database due to its (i) prestige in academia; (ii) professionalism shown in publications to precisely depict the role of EN in the humanities and social sciences in China. Furthermore, CSSCI is known for granting access to high-quality literature with a consistent format, enabling stratified search functions, and having satisfying compatibility with CiteSpace.

Phase 2. Determination of Search Terms and Time Range. A range of phrases considered the variants of the subject term, “Social Network of Enterprise” was used to enrich the data. Apart from “Enterprise Network,” different combinations of keywords including “Enterprise C Social Network” “Enterprise Relational Network” and “Enterprise Internal Network” were incorporated into the period from “1998 to 2020” and it was executed on 01/03/2021. Having excluded literature irrelevant to EN or beyond the research scope of this study, a total number of 603 papers were selected as the sample for the subsequent analyses.

Phase 3. Data Export. The bibliographical records of the retrieved papers were downloaded from the CSSCI database and exported in a plain text format. Then, through CiteSpace’s data conversion function, the exported texts were converted into a format recognizable to CiteSpace.

Phase 4. Data Analysis Using CiteSpace. A bibliographical analysis was conducted based on a valid sample of 603 CSSCI papers, collected through the research mentioned above. First, the converted texts were imported into CiteSpace, and the time-slicing was set as 1998–2020. Second, different node types such as cited author, citation source, and keywords were selected. To be specific, (i) the status quo of EN studies in China was reflected by statistically evaluating the publication time, source journals, researchers, and institutions of sample papers; (ii) the knowledge base of EN studies in China was revealed by establishing knowledge maps based on the citations of sample papers; (iii) the research focus was synthesized, using the keyword-based co-occurrence knowledge map; and (iv) the research trends were revealed through the keyword-based temporal knowledge map. Notably, prior studies could ascertain the validity of these techniques, and they all yielded peer-reviewed results (Olawumi and Chan, 2018).

## Results

In this section, EN-centric research was summarized and synthesized by analyzing the basics of the collected literature, the co-citation analysis, and the co-occurrence analysis of keywords. Analyzing the basics of sample papers, which embodied frequency counts on the volume of publications, key journals, key researchers, and critical institutions, contributed to evaluating the status quo of EN research in China. The co-citation analysis instructed toward authors, sources, and studies frequently cited by sample papers allowed unpacking the knowledge base in the field of EN in China. The co-occurrence analysis of keywords formed the research focus of EN research in China. Finally, the temporal analysis based on the extracted keywords of sample papers enabled the summary of evolutionary trends of Chinese EN studies.

### Status Quo of Enterprise Network Studies in China

#### The Volume of Publications and Source Journals

**FIGURE 2:**
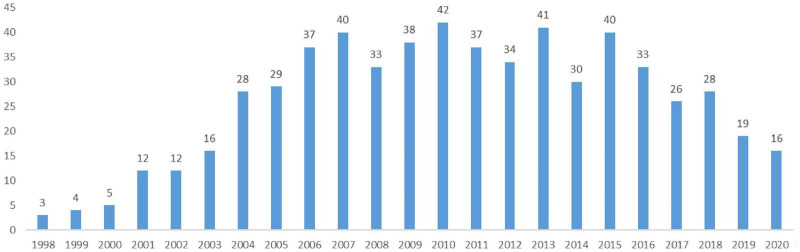
The number of EN papers published in China (1998–2020).

The volume of papers and their annual change in some research areas are pivotal to assessing the academic standards and features displayed during progression, hence estimating the variation tendency of this particular area (Qiu, 2007). Figure 2 demonstrates the number of Chinese EN papers published between 1998 and 2020. According to the bar chart, the number of EN papers has increased since 1998, peaked in 2006 and 2015, and reduced in 2016. Noticeably, although the number is on the decline over time, comparatively many EN papers have been published each year since 2016.

Further, the source journals of sample papers have been statistically evaluated. Results show that 170 CSSCI source journals published at least one paper pertinent to EN. Nonetheless, as is listed in Table 1, only 14 journals have published more than 10 EN papers (Table 1). Even in these journals, the number of corporate social network papers accounts for only a small part of the total number of papers.

**TABLE 1:** The number of EN papers published in CSSCI journals (No. >10).

Journal title	No. of EN papers published	Year of establishment	Total number of articles published in the journal
Science & technology progress and policy	49	2003	14,489
Science of science and management of S. & T.	30	2001	6,998
Science research management	25	2002	4,932
China industrial economics	17	1999	3,973
Soft science	16	2004	5,528
Studies in science of science	16	2007	4,962
Science and technology management research	16	2004	16,232
Business management journal	15	2004	7,755
East China economic management	14	2010	4,749
Productivity research	13	1999	12,871
Chinese journal of management	13	2010	3,215
Foreign economics and management	12	2001	2,533
Nankai business review	11	2004	2,370
Management review	10	2009	3,391

#### Researchers and Institutions

A total number of 968 researchers have participated in EN studies in China. 809 have only written or co-written one paper, accounting for 83.6% of the total amount. According to Price’s Law (1962), taking a further step, the formula for calculating the minimum number threshold for core researcher publications is $T=0.749\times \sqrt{P_{m\,a\,x}}$, which *P*_*max*_ represents the maximum number of publications. The statistics show that the maximum number of publications is 17 (i.e., these 17 papers were written or co-written by Prof. Peng Huatao from Management School, Wuhan University of Technology). After calculation, the minimum number threshold for core researcher publications is 3.09. Thus, researchers with no less than 4 EN publications are considered the core researchers of EN in China (see Table 2 for details). They have 125 papers published collectively, occupying 20.7% of sample papers.

**TABLE 2:** Core researchers of EN studies in China.

Name	No. of publications	Year of 1st publication	Name	No. of publications	Year of 1st publication
Peng Huatao	17	2004	Zhu Haijiu	4	2000
Zhou Lixin	15	2004	Chen Chuanming	4	2004
Liu Dong	13	2002	Zhang Zigang	4	2004
Sun Guoqiang	9	1999	Zhou Xiaohu	4	2004
Zhang Jie	6	2006	Tao Haiqing	4	2005
Mu jifeng	5	2001	Luo Zhe	4	2006
Feng zongxian	5	2001	Peng Zhengying	4	2007
Li Xiaojian	5	2002	Fu Zhengping	4	2008
Liu Bing	5	2005	Dong Baobao	4	2010
Hu Ping	5	2010	Li Feixin	4	2012

For research institutions, the initial step was to normalize the institutions in which sample papers reside. Specifically, the former name of institutions (i.e., institutions that have changed their names at least once) was updated to the latest name to eliminate ambiguity. 405 research institutions have been involved with EN studies after normalization. Unsurprisingly, 277 institutions have only one EN paper published in CSSCI journals. Despite these inactive institutions, Table 3 displays profiles of research institutions with no less than 10 papers published. Given what is presented in Table 3, The Management School of Xi’an Jiaotong University ranks first by producing 21 EN papers. Three institutions share a close second: Management School of Wuhan University of Technology, Business School of Nanjing University, and Management School of the Zhejiang University, as each of them have 19 papers published. In a nutshell, the institutions listed in Table 3 are the pivot for undertaking studies and producing knowledge of EN in China.

**TABLE 3:** Core research institutions of EN studies in China.

Name	No. of publications	Year of establishment
Management school of xi’an jiaotong university	21	2001
Management school of wuhan university of technology	19	2002
Business school of nanjing university	19	2002
Management school of zhejiang university	19	2002
Management school of jilin university	14	2004
Business school of central south university	12	2008
Research centre for economy of upper reaches of the yangtse river at chongqing technology and business university	11	2007
School of economics and management at DUT (Dalian University of Technology)	11	2001
School of economics at zhejiang university	10	2000
Management school of sun yat-sen university	10	2007
Management school of tongji university	10	2009

### Knowledge Base of Enterprise Network Studies in China

#### Analysis of Authors Cited

**FIGURE 3:**
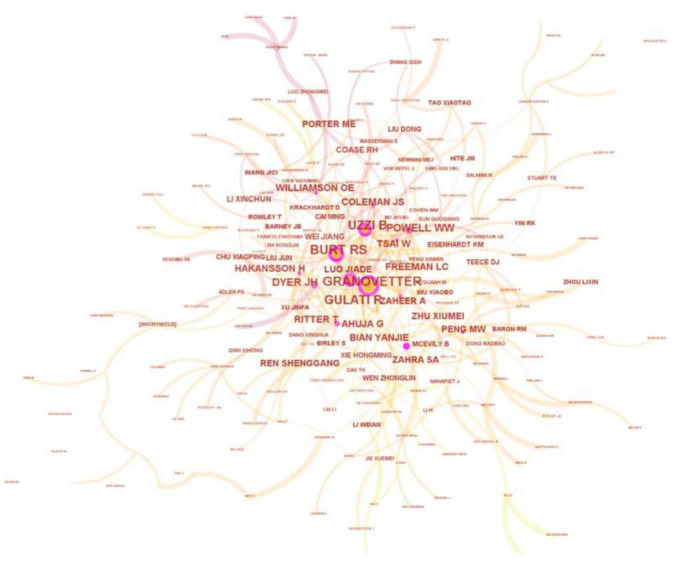
Authors with high frequency of co-citation.

Through analyzing authors that sample papers have extensively cited, key knowledge producers in the field of EN can be discovered. To be specific, CiteSpace was employed to draw a graph of authors with a high frequency of co-citation, as shown below in Figure 3. The entire graph contains 205 nodes and 324 edges, and it is compactly structured as its density is 0.0155. As is displayed in the graph, nodes representing prestigious scholars such as Granovetter, Burt, Gulati, Uzzi, and Coleman are relatively larger size-wise than the others. Hence they are placed in the center.

For a better interpretation of the high author co-citation map, profiles of associated scholars (i.e., co-cited authors as presented in Figure 3) are exhibited in Table 4. Mark Granovetter is the most co-cited author (i.e. 125 times). He suggested theories of “embeddedness” and “weak ties”, introducing fundamental studies of EN’s impact. He also clarifies how these impacts influence enterprise decision making and their financial performance. The second most co-cited author is Ronald Burt (i.e., 96 times), who put forward the conception of “structural holes.” This renowned conception suggests that an individual’s social capital is dictated by intermediate opportunities (i.e., such an intermediate can be perceived as having structural holes). Hence the number of structural holes an individual has is positively related to his/her social capital. Further, Ranjay Gulati’s research about EN and strategic alliance also paved the way for many subsequent studies in the same field. It is noteworthy that apart from foreign scholars, domestic researchers such as Luo Jiade, Bian Yanjie, and Wei Jiang have already gained a strong foothold in shaping the knowledge base of EN in China.

**TABLE 4:** List of authors with high frequency of co-citation.

Author	No. of times co-cited	Half-life period*	Author	No. of times co-cited	Half-life period
Granovetter	125	12	Zahra SA	27	3
Burt RS	96	10	Porter ME	27	7
Gulati R	94	10	Luo Jiade*	25	4
Uzzi B	74	10	McEvily B	23	8
Powell WW	50	9	Bian Yanjie*	23	2
Dyer JH	48	9	Wei Jiang*	23	5
Williamson OE	41	8	Zaheer A	23	7
Coleman JS	41	11	Coase RH	22	3
Ahuja G	39	9	Li Xinchun*	22	6
Tsai W	35	9	Zhu Xiumei*	21	3
Freeman LC	31	9	Wang Jici*	20	6
Ritter T	30	2	Eisenhardt KM	20	7
Hakansson H	29	6	Cai Ning*	20	4
Peng MW	28	8	Ren Shenggang*	20	4

*Half-life period (HLP) was computed by CiteSpace. The higher HLP value is, the more enduring an author’s work is (i.e., the less likely an author’s work is outdated content-wise) (Luo, 1994).

*Names marked with asterisks are Chinese scholars, the others are foreign.

#### Analysis of Sources Cited

**FIGURE 4:**
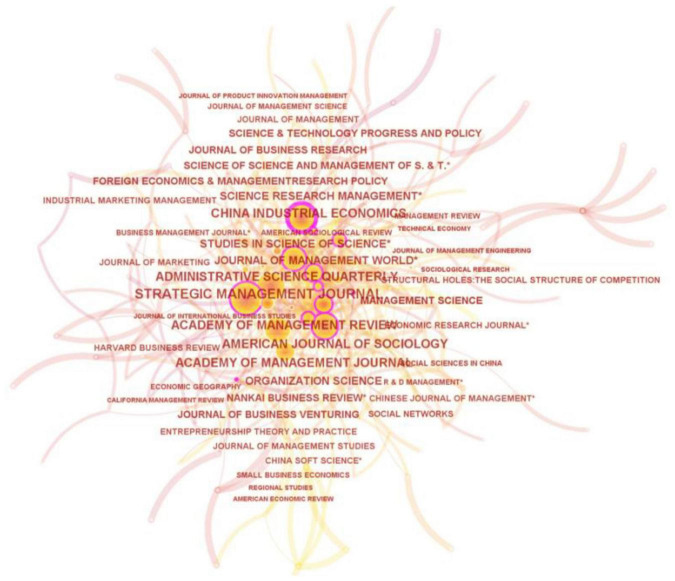
Sources with high frequency of co-citation.

The primary source and key disseminators of knowledge can be found by analyzing sources (e.g., journals, academic institutions, and conferences) widely cited by sample papers. We used CiteSpace to create a map of sources with a high co-citation frequency. This map, containing 237 nodes and 622 edges, is presented in Figure 4.

Noticeably, journals represented by the relatively larger nodes mainly reside in the area of management studies, given that the size of nodes in the map denotes the frequency of citation. Table 5 showcases the 32 most frequently cited sources. Apart from Burt’s work (i.e., “Structural Holes: The Social Structure of Competition”), the other 31 sources are journals, including 18 foreign and 13 domestic ones. English journals with a high frequency of citation such as *Strategic Management Journal*, *American Journal of Sociology*, and *Academy of Management Review*. Alongside Chinese journals that are extensively cited such as *China Industrial Economics*, *Journal of Management World*, and *Studies in Science of Science*, are perceived as the primary sources and critical disseminators of basic knowledge of EN in China. Foreign journals such as *Strategic Management Journal*, *American Journal of Sociology*, and *Academy of Management Review*, and domestic ones such as *China Soft Science*, *Journal of Management World*, *China Industrial Economics*, and *Nankai Business Review*, have a high half-life period (HLP) value covering comparatively more fundamental, indispensable research outcome of EN.

**TABLE 5:** List of sources with high frequency of co-citation.

Source of literature	No. of times cited	Half-life period*	Source of literature	No. of times cited	Half-life period
Strategic management journal	258	12	Industrial marketing management	59	10
American journal of sociology	168	10	Science & technology progress and policy *	58	7
Academy of management review	166	9	Economic research journal*	56	8
Administrative science quarterly	164	10	Journal of management	55	11
Academy of management journal	158	10	Harvard business review	54	10
China industrial economics*	151	10	Journal of marketing	51	6
Journal of management world*	134	11	Journal of management studies	49	10
Studies in science of science*	118	7	Management science	48	6
Organization science	116	11	China soft science*	47	12
Science research management*	100	9	Entrepreneurship theory and practice	47	5
Journal of business venturing	92	10	Structural holes: the social structure of competition	45	6
Research policy	90	10	Social networks	44	6
Nankai business review*	88	10	American sociological review	43	8
Journal of business research	77	7	R & D management*	41	6
Foreign economics and management*	74	8	Chinese journal of management*	39	5
Science of science and management of S. & T.*	73	9	Business management journal*	31	6

*Half-life period (HLP) was computed by CiteSpace. The higher HLP value is, the more enduring a source is (i.e., the less likely a source is outdated content-wise) (Luo, 1994).

*Sources marked with asterisks are Chinese journals, the others are foreign.

#### Analysis of Literature Cited

In general, extensively cited literature echos the inherent nature of the research focus in a research area. Hence, they are often the source of core conceptions that pervade a particular area. In this case, sample papers have 8,482 references in total, among which 7,239 are only cited once. Table 6 demonstrates 15 publications that are most frequently cited by sample papers. As shown in Table 6, the knowledge base of EN in China originates from the propositions associated with social networks, social capital, and EN made by scholars overseas.

**TABLE 6:** List of literature with high frequency of citation.

Author	Title of literature	Journal\publisher	Year of publication*	No. of times cited
Granovetter	Economic action and social structure: The problem of embeddedness	American journal of sociology	1985	69
Burt	Structural holes: The social structure of competition	Harvard university press	N/A	65
Granovetter	Strength of weak ties	American journal of sociology	1973	60
Uzzi	Social structure and competition in interfirm networks: The paradox of embeddedness	Administrative science quarterly	1997	52
Gulati	Strategic networks	Strategic management journal	2000	39
Gulati	Alliances and networks		1998	32
Ahuja	Duality of collaboration: Inducements and opportunities in the formation of inter-firm linkages	Strategic management journal	2000	25
Dyer	Relational View: Cooperative Strategy and Sources of Interorganizational Competitive Advantage	Academy of Management Review	1998	24
Powell	Inter-organizational collaboration and the locus of innovation: Networks of learning in biotechnology	Administrative science quarterly	1996	23
Luo Jiade	Social network analysis*	Social sciences academic press	N/A	21
Xu Jinfa	Analysis of enterprise network competence*	Foreign economics and management	2001	20
Ritter	Network competence: Its impact on innovation success and its antecedents	Journal of business research	2003	20
Coleman	Social capital in the creation of human capital	American journal society	1988	20
Bian Yanjie	Enterprise social capital and its efficacy*	Social sciences in china	2000	17
Powell	Neither market nor hierarchy: Network forms of organization	Research in organizational behavior	1990	17

*Since different editions of a book were merged as one during computation, the year of publication is omitted to avoid potential ambiguity.

*Titles marked with asterisks are Chinese articles, the others are foreign.

Regardless of their development within the scope of sheer sociology, social network theories have been extended to the area of business management studies. For instance, in the well-known work “Economic Action and Social Structure: The Problem of Embeddedness,” Mark Granovetter pinpointed how social relations impacted individuals’ economic behaviors, utilizing the pioneering terminology—“embeddedness” (Granovetter, 1985). This term can be explicated as individuals being “embedded” within their social networks, and thus they are subject to the influence of their social contacts when making decisions. In a similar vein, enterprises, especially when it comes to decision-making and performance, are inevitably affected by their inter-organizational relations. In short, the conceptualization of “embeddedness” interpret the social network between sociological and management studies. An additional effort made by Granovetter—the discussion over the term “weak ties” (Granovetter, 1985)—has also been deemed one of the theoretical building blocks to depict information circulations of EN.

The concept of “social capital” was first put forward by French socialist Pierre Bourdieu in 1977 (Bourdieu, 1977a,b), further extended by James Coleman, proclaiming that social capital can be defined as resources acquired from a specific social structure (Coleman, 1988). Thus, as comprised of integral elements of the social structure, social capital could benefit individuals within this social structure. Additionally, Ronald Burt construed that an individual’s social capital is subject to intermediate opportunities, and therefore, the more structural holes an individual has, the greater his/her social capital is (Burt, 2000).

Moreover, Ranjay Gulati, in his acclaimed publication, “Alliances and Networks,” pointed out that enterprises were positioned within inter-organizational networks, where they constantly interacted with external parties, such as suppliers, customers, and rivals (Gulati, 1998). Such interactions were no longer bilateral, meaning that they were networked for multiple allied parties to obtain reciprocity. The scholar emphasized that “Strategic Networks” would effectively benefit an enterprise to acquire information, resources, marketplace, and technology. Further, it could achieve strategic goals such as outsourcing value chains, sharing risks, and ultimately enhancing competitive advantages through organizational learning, economies of scale, and economies of scope (Gulati et al., 2006).

Aside from the foreign literature discussed above, some domestic papers have been frequently cited. The leading article, “Analysis of Enterprise Network Competence,” written by Xu et al. (2001), plays a crucial role in the field. The authors firstly defined “Enterprise Network Competence” (ENC) as an enterprise’s capacity to govern and advance its external social networks, secondly revealed the nature and holistic structure of ENC, and finally made implications regarding how ENC could be improved (Xu et al., 2001). Further, Bian and Qiu (2000) in their paper, “Enterprise Social Capital and Its Efficacy,” offered new insights into the concept of “Enterprise social capital.” To be specific, they categorized an enterprise’s effort to gain social capital into three types: (i) vertical ties (i.e., an enterprise’s connection with government or other authorities); (ii) horizontal ties (i.e., an enterprise’s connection with others across different sectors); and (iii) social ties (i.e., the proprietor’s contacts) (Bian and Qiu, 2000). The well-being of an enterprise’s social capital is dictated externally by the economy’s structure and activated (Bian and Qiu, 2000). Hence, vertical (i.e., whether enterprise representatives used to work in any of the relevant authorities), horizontal (i.e., whether enterprise representatives used to work in the top management of any enterprises across different sectors) and social ties (i.e., whether enterprise representatives have substantial social contacts) were the three indicators to gauge an enterprise’s social capital (Bian and Qiu, 2000). Additionally, the methods introduced by Luo Jiade in his milestone work “Social Network Analysis,” that were specified for social network analysis have been seen as an integral part of the knowledge base in EN (Luo, 2010).

Considering the analysis above, EN-centric studies in China have primarily drawn upon prior works published in English journals. Most of the core conceptions were proposed by non-Chinese scholars such as Granovetter, Burt, Gulati, Uzzi, and Coleman. Nonetheless, it has been witnessed that domestic researchers such as Luo Jiade, Bian Yanjie, and Wei Jiang have made endeavors to extend existing theories by incorporating distinct features of business prospects and enterprise performance in China. In other words, the research outcome of these domestic scholars is part of the knowledge base of EN in China.

### Hot Topics of Enterprise Network Studies in China

To further explore the hot topics and potential future topics, we conducted a co-occurrence analysis on keywords by using CiteSpace. A total of 1,301 keywords were collected after the normalization process (e.g., merge terms having identical connotations).

#### Statistics of High-Frequency Keywords

Among the 1,301 keywords collected from sample papers, 1,049 keywords have only emerged once, accounting for 80.6% of the population. Table 7 lists keywords that have been mentioned no less than 10 times, and these terms are seen as the high-frequency keywords in this part. Aside from the search terms (e.g., “Social Network of Enterprise” and “Social Network”), which were used for collecting sample papers, the other keywords displayed in Table 7, such as “industrial cluster” “innovation performance” and “SME” (i.e., Small and Medium-Sized Enterprise), represent, to a large extent, the scholarly interest in the area of EN in China.

**TABLE 7:** List of high-frequency keywords.

Keyword	Frequency	Year	Keyword	Frequency	Year
Enterprise network	195	1998	Organization of enterprise network	15	2001
Social network	121	2004	Start-ups	14	2005
Network competence	32	2007	Social network analysis	11	2005
Industrial cluster	30	2004	Entrepreneurs	11	1999
SME	22	2000	Competitive advantage	11	2002
Innovation performance	22	2010	Technological innovation	10	1998
Social capital	20	2000	Knowledge sharing	10	2005
Enterprise performance	20	2008	Network position	10	2009
Family business	19	2003	Enterprise growth	10	2002
Network structure	17	2005			

#### Co-occurrence Analysis on Keywords

**FIGURE 5:**
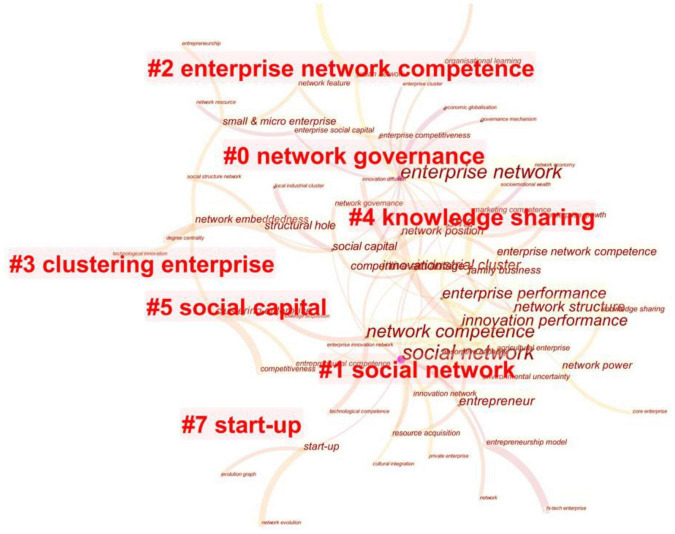
Clusters of high-frequency keywords.

By compiling, categorizing, and merging the seven clusters divided by CiteSpace (Figure 5), we obtained a network consisting of conceptualization of EN, EN as Indicators of Enterprise Development, EN’s Impact on Start-Ups, mechanisms of EN’s effect and governance of EN five hot topics.

The hot topic “conceptualization of EN” mainly concentrates on the EN’s theoretical connotations and effect, the earliest EN research topic in China. Due to the rapid growth of information-related industries, the interplay and collaboration among enterprises were greatly strengthened (Zhen, 1998). Under such a circumstance, scholarly attention in China was directed to inter-enterprise relational networks (Huang and Niu, 1999). The enterprises’ correlation of diverse perspectives includes “strategic alliances” (Sun and Lan, 1999), “virtual enterprises” (Zhang and Gan, 1999), and “organization of enterprises” (Chen, 2000). Also, from a methodological point of view, social network analysis began to be employed by many to materialize the effect of inter-enterprise relational networks, given the introduction of the acclaimed EN concept—“embeddedness.” Further, social capital became an exciting research term since discussions over its definition and usage were extensive. For instance, Bian and Qiu (2000) contended that enterprises were nodes embedded in networks of multiple forms rather than isolated entities. Social capital was intimately associated with the relational networks established by enterprises. Moreover, it was inherently beneficial to enterprises. They used surveys and structural equation models further ascertain the relation between EN and social capital (Sheng, 2010). The researcher quantitatively pinpointed how social networks facilitated the acquisition of social capital from five aspects: network scale, network heterogeneity, network position, the strength of ties, and durability of ties (Sheng, 2010). Initially, entrepreneurs’ social networks appealed to researchers (Xue and Tao, 2004; Liu et al., 2005; Yang and Huang, 2005), who found out that social networks mainly shaped by entrepreneurs’ ties helped sustain the corresponding new ventures. As relevant research progressed further, the scope of this research topic was extended. The emphasis was no longer confined to entrepreneurs’ social networks as inter-organizational networks were increasingly exploited (Shi, 2012).

The hot topic “EN as Indicators of Enterprise Development” is the main body of EN research in China. From a resource dependency perspective, an enterprise’s growth is mainly subject to the resources it can garner. Since EN has been widely accepted as the critical source of external resources, gauging the number of network resources and its influence over enterprises’ strategic deployment, innovation performance, and mergers & acquisitions performance has been the most exciting themes among scholars in China. Specifically, the indicators for network positions within a social network are the observed variables to gauge such resources, and they mainly include centrality and structural hole (Zhu et al., 2017). Here, centrality primarily refers betweenness centrality, betweenness, and eigenvector centrality. Closeness centrality is often excluded, for most researchers cannot garner completely connected social networks. As a result, the value of such indicators can generally reflect pertinent advantages gained by enterprises within a social network (Wang and Xiong, 2020).

Further, in an independent director network, the centrally positioned ones are thought to effectively expedite mergers and acquisitions and augment follow-up performance by providing consultation and other information services (Liang et al., 2018). Apart from the centrality mentioned above, indicators intended to symbolize the prestige within a network, Chinese researchers frequently employ the structural hole to measure an enterprise’s information and control superiority. When an enterprise achieves a relatively higher value of structural-hole indicators, its operational and investment efficacy will improve (Guo and Xie, 2020). Although centrality is often linked to the structural hole, they differ in how an enterprise can profit from them. Li et al.’s work ascertained that enterprises with higher centrality values (i.e., centrally positioned enterprises) outperformed those with more structural holes in procuring information and resources (Li et al., 2017).

The hot topic “EN’s Impact on Start-Ups” is how start-ups acquire external resources from their social networks. Compared with large corporations, start-ups are more often confronted with threats to survival, such as limited funds and resources for expansion. It was affirmed that social networks had been deemed a key element to the survival and growth of new ventures. They functioned to obtain resources, reduce uncertainty, and establish a reputation (Rui et al., 2020). A similar finding was yielded in Zhu and Li (2011). They found that start-ups favored their social networks for raising funds in a transitional economy where the institutional trust was low (due to unsound government functions, inadequate law system, and inefficient financial system) (Zhu and Li, 2011). The finding was later ascertained by Chen et al. (2015) with a sample of start-ups in China’s private sector, classified social networks as personal, business, institutional, and governmental networks. All four types asserted that social networks could facilitate a start-up’s operational performance. More recent studies shed new light on the topic. Xiao et al. (2018) statistically affirmed that the extent to which a start-up is embedded in its social network is positively related to its business prospect. Finally, Liu et al. (2016) pointed out that social networks also helped boost empolyees’ morale, which eventually solidified the venture’s development.

The hot topic “Mechanisms of EN’s Effect” explores how the resources impact enterprise growth and how the organizational factors help facilitate such an impact. Here, the extent to which these resources were assimilated and utilized was key to the impact of network position on enterprise performance (Qian et al., 2010). Qian et al. (2010) empirically ascertained that only enterprises with a robust capacity for acquiring and assimilating knowledge were able to be promoted to a higher network position. They were exposed to ampler innovation-related returns. Furthermore, Feng theorized the capacity mentioned above as network competence claiming that enterprises with strong network competence were more likely to benefit from networks (Feng, 2010). Shi et al.’s survey-based study yielded a similar result (Shi et al., 2014). The study confirmed that social networks mainly influenced an enterprise’s capacity for technological innovation using assimilation and conversion of knowledge (Shi et al., 2014). Finally, Ren classified EN competence as network vision, network construction, relation management, and relation combination (Ren, 2010). The scholar also assessed the correlation between EN competence and innovation performance by adopting a structural equation model. The result suggested that the former facilitated the latter significantly, and the strength of ties and network position functioned as intermediaries in between (Ren, 2010).

The hot topic “Governance of EN” is centered around pondering the cause and effect of EN’s positive and negative impact. Obtaining resources from their social networks to reinforce competitiveness is essential for enterprises. Enterprises are increasingly engaged in trade and manufacturing through inter-organizational coordination. In other words, enterprises are progressively “embedding” themselves into social networks to facilitate self-improvement. Nonetheless, the impact of social networks on enterprise growth is occasionally negative (Liu, 2016). Hence, it becomes imperative to delve into how the negativity brought by social networks can be mitigated, and this is deemed the focal point of this research topic. When it comes to specific literature, Zhou described EN’s negative influence as “enterprise social liabilities,” a threefold concept that embodies cost of resource acquisition, risk of network-based selection, and negativity of network resources (Zhou and Chen, 2005). These three facets guide optimizing network resources and eschewing social liabilities. Governance is considered vital in enterprise alliances bound by economic contracts and social relations. The purpose of governance is mainly to coordinate and maintain each enterprise within the alliance to optimize internal and external resources (Zhou and Chen, 2005). Here, Xu stressed that the governing models and mechanisms varied across different forms of alliances (Xu, 2010). For instance, governance largely relies on power in market-driven alliances, whereas it heavily depends on social capital in strategy-driven alliances (Xu, 2010). More importantly, EN, acting as the systematic deployment of organizations, takes different forms other than alliances. From the viewpoint of new economic sociology, enterprises’ behaviors are also embedded in their social networks. In this case, Zeng asserted that the network governing mechanisms are deeply rooted in (i) mutual dependency of network members; (ii) cost of network coordination; and (iii) opportunistic cutting-down (Zeng, 2010). Additionally, given the rapid growth of e-commerce, Li and He pointed out that the existing governing mechanisms varied under e-commerce in China (Li and He, 2013). They also stressed that relational contract and rapport among enterprises remained the cornerstone of integrating network resources (Li and He, 2013).

### Research Trends of Enterprise Network Studies in China

**FIGURE 6:**
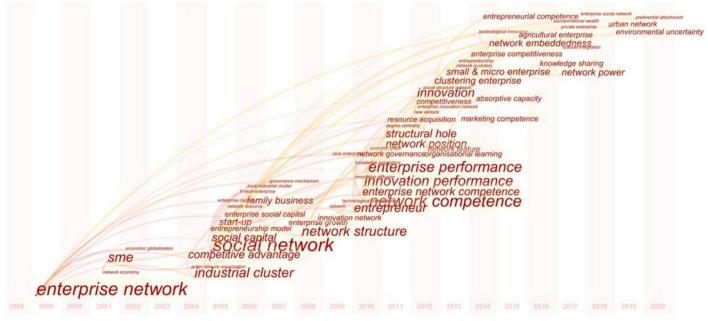
Temporal knowledge map of high-frequency keywords.

To paint a complete picture of the development trajectory of EN in China, CiteSpace was used again to extract high-frequency keywords each year between 1998 and 2020 and draw a temporal knowledge map (i.e., Figure 6) accordingly. This temporal analysis, coupled with the publication time and quantity of papers (i.e., from which the analyzed high-frequency keywords were extracted), divided the progression of EN in China into two phases: (i) preliminary stage (1998–2007); and (ii) growth stage (2007–2020). These two phases are elaborated as follows.

#### Preliminary Stage (1998–2007)

During this period, Chinese researchers started to draw upon EN theories from their peers overseas. As the co-opted EN theories were contextualized (e.g., features of China’s business environment), a burst of publications (i.e., continuous growth from 1998 to 2007) could be observed. The focal point in this stage shifted from introducing existing concepts and frameworks to the localization of such theories. Keywords extensively used in this stage included “Enterprise Network,” “Network Economy,” “Industrial Cluster,” “Social Capital,” “Start-Up,” and “Enterprise Growth.” Papers published in this stage enhance EN’s role in the following aspects: enterprise growth, sustainability of virtual enterprises, collaborative innovation of technologies, and competitive advantages.

#### Growth Stage (2007–2020)

According to Figure 1, the volume of EN publications in China has ceased to increase rapidly and started to undergo an erratic fluctuation since 2007. Similarly, in Figure 6, many new keywords emerged after 2007. As EN research in China has become more well-known; thus, terms such as “Network Structure,” “Network Position,” “Structural Hole,” “Enterprise Competitive Advantage,” “Enterprise Performance,” and “Network Competence” have been used more frequently. The focal point is mainly associated with EN’s impact on enterprises’ endeavors to gain competitive advantages, embrace innovation, enhance performance, and govern social networks to maximize profitability. In this period, scholars primarily emphasized how enterprises (especially SMEs and start-ups) efficaciously utilize their social networks. That is, how to tackle the problems emerging from the formation of industrial clusters, mergers and acquisitions, and materialization of innovation and eventually improve organizational performance. Additionally, high-frequency keywords such as “Urban Networks” indicate that new insights and perspectives have been consistently embodied in EN studies in China.

In short, given the temporal analysis of high-frequency keywords, as theories pertinent to social networks have been introduced and contextualized with practice in China, there is a clear sign of localization for EN research in China. The localization of concepts and frameworks of EN successively embark on EN theories to interpret competitiveness-increasing mechanisms found in areas including industrial clusters and start-ups, and finally adopting empirical approaches to unveil EN’s impact on innovation, mergers and acquisitions, conducts and performance, from the viewpoint of structure and competence. It indicates that EN research in China has upgraded to a more sophisticated level. In terms of the ongoing trend in EN, it is evident that the combination between EN and other forms of networks, including geographical and knowledge networks, is potentially becoming the focus of future studies.

## Discussion and Conclusion

In China, EN is a topic that numerous articles have shaped for years, but the understanding of current research remains chaotic. This paper researches and uncovers the development of Enterprise-Network’s studies from 1998 to 2020 in China by bibliometric analysis and provides comprehensive, quantitative reviews exclusively focused on EN.

This study collected 603 papers from the CSSCI, and the time range covers the period from 1998 to 2020. Over time, the distributions of EN papers show accelerated growth starting from 2006, resulting from the increasing number of works on EN in various scientific fields and disciplines applying EN in their studies (Zhou and Ruan, 2018; Maltseva and Batagelj, 2020). One hundred seventy CSSCI source journals have published EN papers, but only 14 of them have published more than 10 EN papers. In these 14 journals, papers on EN only account for a very small proportion. As many as 83.6% of authors wrote or contributed to only one paper in the sample literature. As Lotka’s Law suggests, the number of authors with only one paper approximately occupies 60% of the total in typical situations. It implies that most researchers only have a provisional interest in EN, and the core research clusters for EN are relatively limited.

Our analysis has gained a basic understanding of Chinese EN research through the knowledge graph of citations. The works concerning social networks, relational embeddedness, social capital, and structural holes, written by western scholars including Granovetter, Burt, Gulati, and Coleman, alongside domestic scholars including Luo Jiade, Bian Yanjie, and Wei Jiang, have collectively formed the knowledge base of EN in China. It can be seen that although the EN theory originated from the Western scenario, concepts such as enterprise social capital (Bian and Qiu, 2000) and EN capability (Xu et al., 2001) proposed by Chinese scholars based on the characteristics of the Chinese market and system have formed the knowledge base of EN research. Among the highly cited journals, in addition to Social Networks, professional journals in the fields of sociology, management, and organizational science are often cited as knowledge sources for EN. It coincides with part of the conclusions reached by Maltseva and Batagelj (2021), with the difference in that journals in computer science and physics are less cited in Chinese EN research.

The results of Co-occurrence analysis on High-Frequency keywords show that the hot topics of EN in China can be summarized and categorized as follows: conceptualization of EN, EN as indicators of enterprise development, EN’s impact on start-ups, mechanisms of EN’s effect, and governance of EN. Among those, EN as indicators of enterprise development and EN’s impact on start-ups have received the most attention from researchers. Most of the sample literature falls into these two categories. According to Li and Jia (2014) and Maltseva and Batagelj (2020), western research on social networks is mainly conducted from the structural and instrumental perspectives. Based on the analysis of the research focus in this study, current research on EN in China is mainly carried out from the instrumental perspective, that is, to use network relations and network position to explain different issues in corporate management. Only a few scholars have studied the formation mechanism of EN based on the structural perspective. Chang and Wang (2016) found that corporate strategy and financial needs play a significant role in promoting the formation of the EN formed by cross-shareholding relationships. It explains why many temporary EN researchers and computer science and physics journals are less cited in China. Most researchers use EN merely as an explanatory variable to solve problems in corporate management, such as knowledge management, organization performance, and resource acquisition. The focus of research is not on EN itself.

Based on the analysis of research trends, the potential direction for future studies ought to give weight to the integration between EN and other forms of networks, such as geographical networks (Wang and Sun, 2020; Zhang et al., 2020), and the mechanism of action of specific EN, such as the action mechanism of EN for start-ups in the context of environmental uncertainty (Chen and Feng, 2019; Li et al., 2019).

This study shows that research on EN in China has evolved in its complexity and reached remarkable results, but there is still a considerable gap to fill in the research on the structure of EN. In the future, in addition to viewing EN as a tool to explain enterprise organization and management, EN research in China should pay more attention to topics concerning network structure, such as multi-level EN, topology structure of the EN, and the formation path of the EN.

This study’s bibliometric analysis helps portray a comprehensive framework of EN research and enables future scholars to focus on their studies effectively. However, we have to acknowledge that this study also has limitations. First, the sample in our study is captured in only one database. As the most influential literature database in China, the CSSCI only contains high-quality journals in Chinese Social Sciences, so it can not cover all publications on the theme of EN. According to Bradford’s law, most papers in a specific field are published in a few professional journals in this field. Therefore, this paper’s literature samples obtained from the CSSCI database are highly representative. Second, although multiple combinations of terms and phrases were used in search of as many relevant EN papers as applicable, this study is still confined to literature published since 1998 due to the capacity of the CSSCI (i.e., only includes studies from 1998 to the present days). Nonetheless, it is unlikely that the findings are undermined by the exclusion of literature before 1998, as only a little Chinese EN research can be identified in that period. Finally, although the bibliometric analysis employing specialized software is objective, the following interpretation of the results is somewhat subjective. Different researchers have different cognitions and interpretations of even the same content. Through multiple discussions between authors, this study tries to overcome the subjective interpretation from only one researcher.

## Author Contributions

WY and CC: conceptualization, validation, and writing—original draft preparation. WY: methodology, software, investigation, and funding acquisition. SW: resources. CC, WY, HL, QZ, XS, and SW: writing—review and editing. CC: supervision. All authors have read and agreed to the published version of the manuscript.

## Conflict of Interest

The authors declare that the research was conducted in the absence of any commercial or financial relationships that could be construed as a potential conflict of interest.

## Publisher’s Note

All claims expressed in this article are solely those of the authors and do not necessarily represent those of their affiliated organizations, or those of the publisher, the editors and the reviewers. Any product that may be evaluated in this article, or claim that may be made by its manufacturer, is not guaranteed or endorsed by the publisher.
